# Progressive multifocal exophytic pontine glioblastoma: a case report with literature review

**DOI:** 10.1186/s40880-017-0201-z

**Published:** 2017-03-27

**Authors:** Fanfan Chen, Zongyang Li, Chengyin Weng, Peng Li, Lanbo Tu, Lei Chen, Wei Xie, Ling Li

**Affiliations:** 1 Neurosurgery Department, Guangzhou First People’s Hospital, Guangzhou Medical University, Guangzhou, 510180 Guangdong P. R. China; 2 Neurosurgery Department, Shenzhen Second People’s Hospital, Shenzhen University, Shenzhen, 518000 Guangdong P. R. China; 3 Oncology Department, Guangzhou First People’s Hospital, Guangzhou Medical University, Guangzhou, 510180 Guangdong P. R. China; 4 Record Department, Guangzhou First People’s Hospital, Guangzhou Medical University, Guangzhou, 510180 Guangdong P. R. China

**Keywords:** Brainstem, Cerebello-pontine angle, Glioma, Multiple lesion, Pontine

## Abstract

Multifocal pontine glioblastoma exhibiting an exophytic growth pattern in the cerebello-pontine angle (CPA) is rare. We present a case of a 5-year-old girl with consecutive neurological imaging and other clinical findings indicating progressive multifocal exophytic pontine glioblastoma. Three lesions were reported, of which two were initially presented, and one was developed 2 months later. One lesion demonstrated a progressing exophytic extension in the cistern of the left side of the CPA. The other two lesions were located and confined within the pons. Initial magnetic resonance imaging and positron emission tomography–computed tomography indicated low-grade glioma or inflammatory disease. However, 2 and 3 months later, subsequent magnetic resonance spectroscopy (MRS) displayed elevated choline and depressed *N*-acetyl aspartate peaks compared with the peaks on the initial MRS, indicating a high-grade glioma. Subtotal resection was performed for the CPA lesion. Histopathologic examination showed discrepant features of different parts of the CPA lesion. The patient received no further chemotherapy or radiotherapy and died 2 months after surgery. The multifocal and exophytic features of this case and the heterogeneous manifestations on neurological images were rare and confusing for both diagnosis and surgical decision-making. Our case report may contribute knowledge and helpful guidance for other medical doctors.

## Background

Although brainstem tumors account for 10%–20% of cerebral neoplasms in children and 90% of them are gliomas [1, 2], exophytic pontine glioma located in the cerebello-pontine angle (CPA) is uncommon [3–6]. Because of their heterogeneous biological behaviors and neurological imaging manifestations, the classification of brainstem gliomas is not consistent. Diagnosis and therapeutic decision-making are often difficult for gliomas, especially in the case of rare neurological imaging features.

Here, we report a special case of multifocal glioblastoma derived from the pons, exhibiting an exophytic growth pattern, in a 5-year-old girl. In total, three lesions were revealed in the posterior fossa. Repeated magnetic resonance imaging (MRI) showed the progression of the lesions in the left side of the CPA and the upper left side of the pons as well as the appearance of a new lesion in the right side of the pons. Furthermore, repeated magnetic resonance spectroscopy (MRS) over 3 months revealed a gradually elevated ratio of choline (Cho) to *N*-acetyl aspartate (NAA).

## Case report

### Medical history and examination

The 5-year-old girl was admitted to the Neurosurgery Department of Guangzhou First People’s Hospital, Guangzhou, China on October 6, 2015 and presented with a 2 month history of a medial gaze of the left eye. She did not complain of headache, dizziness, dysphagia, or vomiting. The outpatient medical record revealed that the patient frequently had fevers and coughs since the age of three. The last fever and cough were reported 1 week before the admission. The patient showed age-appropriate physical development. Cardiopulmonary auscultation results were normal. A neurological examination revealed left cranial VI nerve palsy without involvement of other nerves such as the ocular motor (cranial III or IV), trigeminal, facial, and acoustic nerves. No cerebellar signs or hemiparesis were present. The patient showed no signs of ataxia or long tract dysfunction.

**Fig. 1:**
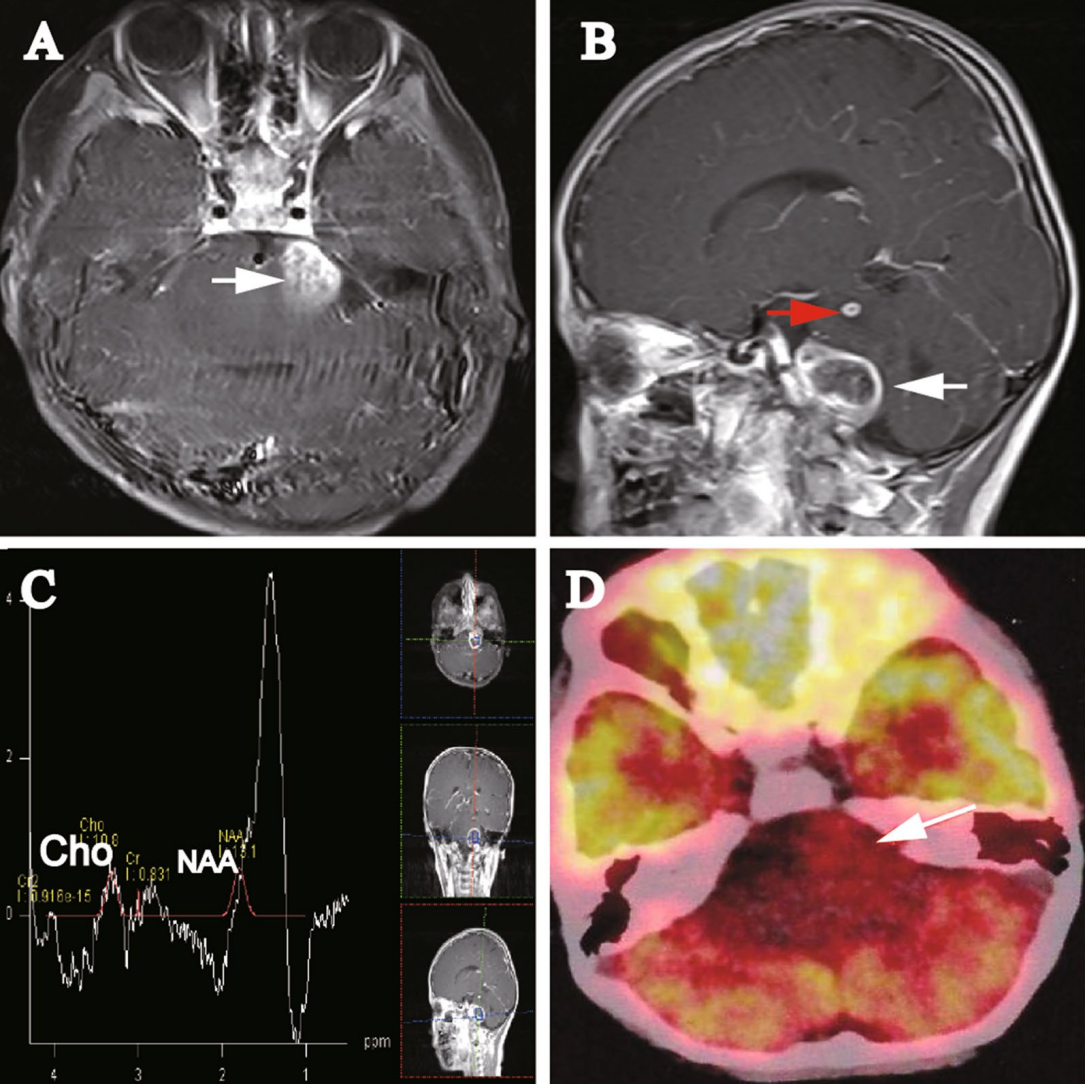
Images of the initial magnetic resonance imaging (MRI) and positron emission tomography–computed tomography (PET–CT) on Oct 8, 2015 for the 5-year-old girl with multiple posterior fossa lesions. **A** Axial gadolinium-enhanced MRI displays a lesion (2.0 cm × 2.6 cm; *white arrow*) in the cerebello-pontine angle (CPA). No relatively clear margin was observed between the lesion and the pons. **B** Sagittal gadolinium-enhanced MRI shows two lesions. The *upper left* pontine lesion (0.5 cm × 0.6 cm) was significantly gadolinium-enhanced with relatively clear margin (*red arrow*). From the sagittal view, the CPA lesion (*white arrow*) seemingly demonstrates clear boundary to the pons. **C** Magnetic resonance spectroscopy (MRS) of the lesion shows a decrease in choline (Cho) and *N*-acetyl aspartate (NAA). **D** PET–CT image demonstrates a hypometabolic lesion (*white arrow*) in the *left side* of the CPA

Gadolinium-enhanced magnetic resonance imaging (MRI) was performed 2 days after the admission, demonstrating two well-defined, ring-enhanced lesions at the left side of the CPA (2.0 cm × 2.6 cm; Fig. 1A) and upper left side of the pons (0.6 cm × 0.5 cm; Fig. 1B). The MRI manifestation of the CPA lesion was similar to an extra-axial mass, e.g., nerve sheath tumor. The pons was obviously enlarged and swollen. MRS displayed a simultaneous decrease in *N*-acetyl aspartate (NAA) and choline (Cho) (Fig. 1C). The Cho:NAA ratio was 0.82, and the Cho:Cr (creatine) ratio was 13.01. A peak at 1.3 part per million (ppm) was observed. Positron emission tomography–computed tomography (PET–CT) was performed, which demonstrated hypometabolic foci in the left side of the CPA, indicating low-grade glioma or inflammation (Fig. 1D).

The patient showed weak expression of serum antibodies for tubercle bacillus (TB). The results of sputum smear examination and blood interferon-gamma release assay for the detection of TB infection were negative. Serum levels of tumor markers [including alpha-fetoprotein, carcinoembryonic antigen, carbohydrate antigen (CA)-724, CA-125, CA-199, and squamous cell carcinoma antigen] were within normal ranges, except for a slight increase in the level of neuron-specific enolase. The cerebrospinal fluid was extracted via lumbar puncture and tested for TB-DNA and TB-RNA, yielding negative results. After consultation with a tuberculosis specialist, the current evidence for intracranial tuberculoma was deemed insufficient. We proposed a biopsy of the left CPA lesion, but the patient’s parents refused and demanded discharge.

**Fig. 2:**
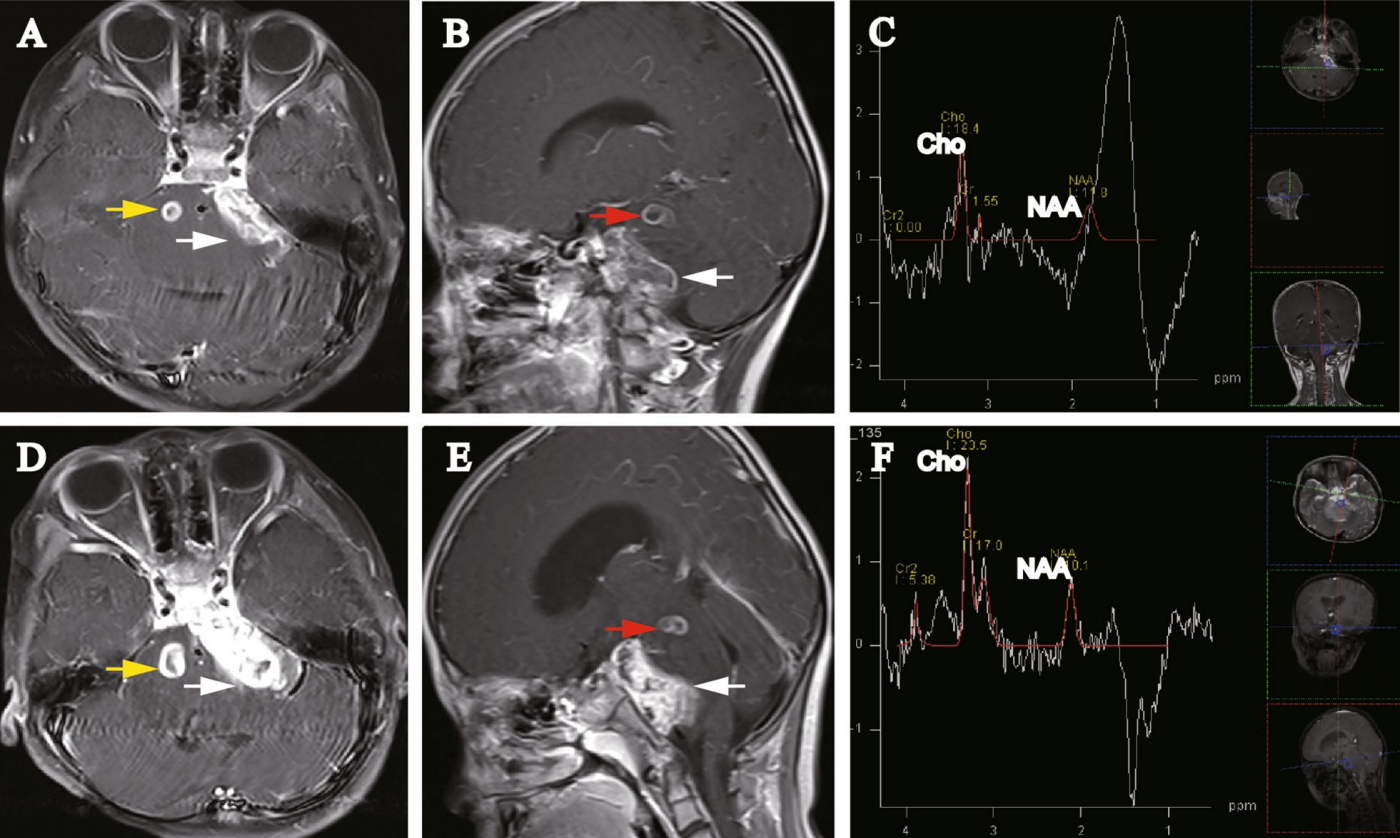
Images of repeated MRI on Dec 11, 2015 and Jan 12, 2016 for the 5-year-old girl with multiple posterior fossa lesions. **A** Axial gadolinium-enhanced MRI (Dec 11, 2015) displays the occurrence of a new lesion (0.8 cm × 0.9 cm, *yellow arrow*). The *left* CPA lesion is enlarged and extended along the cistern of the CPA (3.1 cm × 1.2 cm, *white arrow*). **B** Sagittal gadolinium-enhanced MRI (Dec 11, 2015) shows that the CPA lesion was enlarged and extended along the cistern of CPA (*white arrow*) and the *upper left* pontine lesion was enlarged (*red arrow*). **C** MRS (Dec 11, 2015) shows increased Cho and decreased NAA expression. The Cho:NAA ratio was also significantly increased. **D** Axial gadolinium-enhanced MRI (Jan 12, 2016) displays that both the CPA lesion (*white arrow*) and the new lesion in the *right side* of the pons are enlarged (1.1 cm × 1.3 cm, *yellow arrow*). The CPA lesion is extended along the cistern of the CPA. **E** Sagittal gadolinium-enhanced MRI (Jan 12, 2016) shows that both the CPA lesion (*white arrow*) and *upper left* pontine lesion (*red arrow*) were enlarged. **F** MRS (Jan 12, 2016) shows further increased Cho and decreased NAA expression. The Cho:NAA ratio was 2.33

Two months later, the patient underwent another MRI. No involvement of additional nerves was observed, except for the cranial VI palsy. However, progression of the disease was demonstrated: (1) the left CPA lesion was enlarged and extended along the cistern of CPA (Fig. 2A, B, white arrow); (2) the upper left pontine lesion was enlarged (Fig. 2B, red arrow); and most importantly, (3) a new lesion had emerged in the upper right side of the pons (Fig. 2A, yellow arrow). In addition, MRS revealed a radical decrease of NAA and a drastic increase of Cho with an obvious increase in the Cho:NAA ratio (Cho:NAA = 1.56) and a decrease in the Cho:Cr ratio (Cho:Cr = 11.87) (Fig. 2C). A peak at 1.3 ppm was displayed. We strongly suggested surgery for pathologic diagnosis and follow-up treatment as soon as possible, but the parents refused again.

One month later, the patient was admitted again because of malnutrition and weakness with frequent vomiting. Re-examination with MRI demonstrated progression of all three lesions, especially the CPA lesion, which progressed and expanded to the anterior CPA cistern (Fig. 2D, E). MRS data showed the same pattern as the previous examination, with a significantly increased Cho:NAA ratio (Cho:NAA = 2.33) and dramatically decreased Cho:Cr ratio (Cho:Cr = 1.38) (Fig. 2F). A reverse peak at 1.3 ppm was shown. The parents finally agreed to the suggested surgery.

### Surgery and pathology

**Fig. 3:**
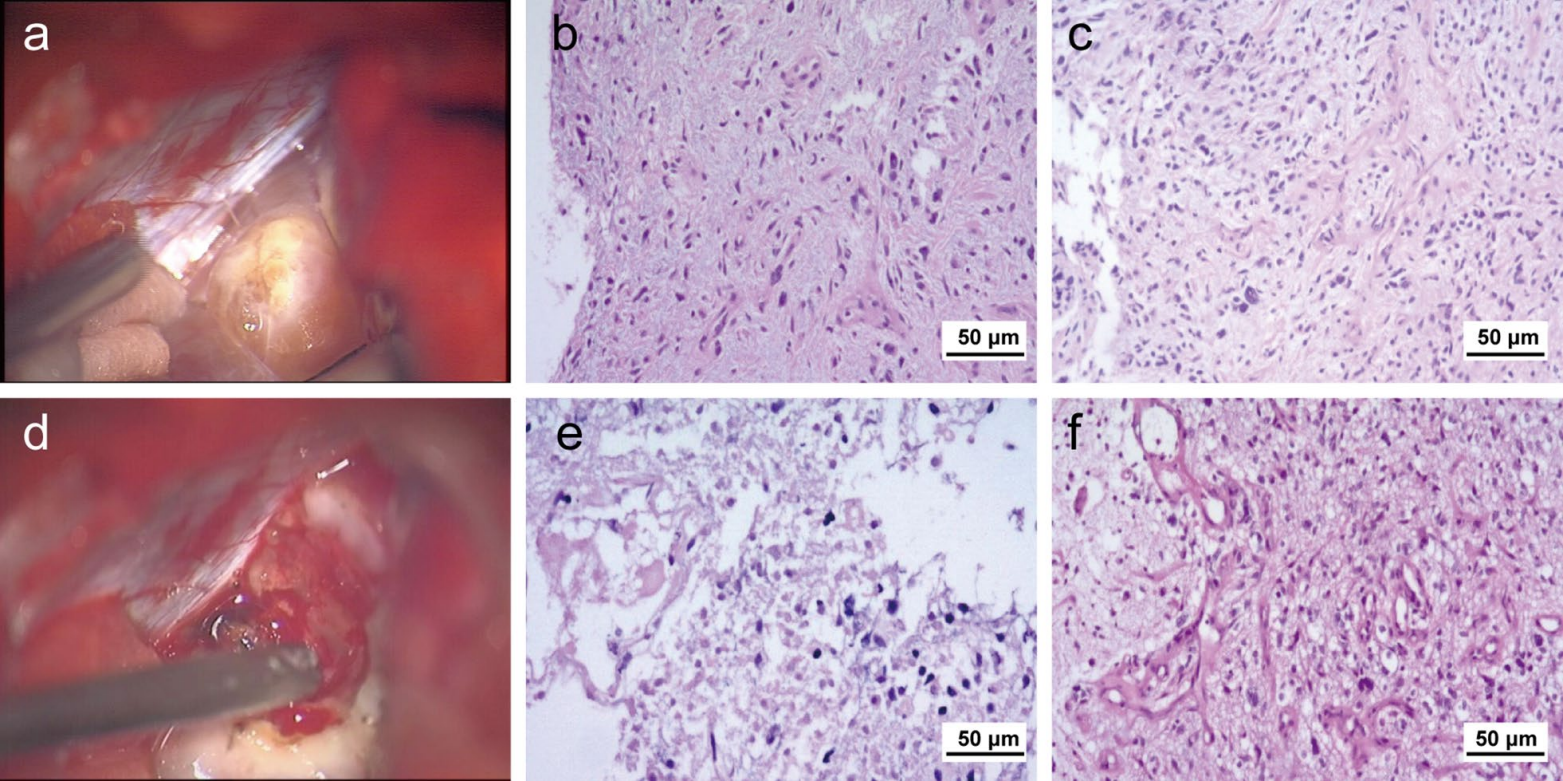
Histopathologic examination of the CPA lesion with hematoxylin–eosin staining. **a** Macroscopically, the outer layer of CPA lesion is rubbery and nodule-like. **b** Under a microscope, the outer layer of the CPA lesion displays moderate mitoses and necrosis. **c** Under a microscope, the outer layer of the CPA lesion displays moderate vascularization. **d** Macroscopically, the internal portion of the lesion is softer and has a higher blood supply than the outer layer. **e** Under a microscope, the internal portion of the CPA lesion displays obvious mitoses and necrosis. **f** Under a microscope, the internal portion of the CPA lesion displays hyper-vascularization

Histopathologic diagnosis and safe resection of the lesion were the goals of the suggested surgery. Under these circumstances, the CPA lesion was the target of the operation, whereas the other two lesions were left untouched. The surgery was conducted using a suboccipital retrosigmoid approach. After occipital craniotomy and dura opening, the cerebellum was cautiously separated from the CPA, and the cerebrospinal fluid was slowly released and suctioned. The arachnoid of the CPA was opened, and a yellowish nodule-like mass was encountered with a clear boundary to the cerebellum (Fig. 3a). The vessels distributed on the surface of the lesion were carefully coagulated, and the lesion was moderately rubbery. The tissue was formalin-fixed and paraffin-embedded for a pathologic examination with hematoxylin–eosin staining, which indicated moderate mitoses, necrosis, and vascularization, suggesting low-grade glioma (Fig. 3b, c). Resection of the lesion combined with ventricular peritoneal drainage was performed. The internal portion of the lesion, with a relatively higher blood supply, was softer than the outer layer (Fig. 3d). No clear boundary was found between the lesion and the pons or the root entry zone of the trigeminal nerve. Histopathologic examination revealed a highly malignant tumor with marked necrosis and vascularization (Fig. 3e, f). No typical pseudopalisading signs or glomerular structures were observed in this case. The internal portion of the CPA lesion was removed within the arachnoid boundary until neurophysiological monitoring of the caudal cranial nerves displayed a slightly decreasing reaction. Postoperative MRI confirmed the subtotal resection of the CPA lesion. No further surgery-related neurological deficits occurred.

**Fig. 4:**
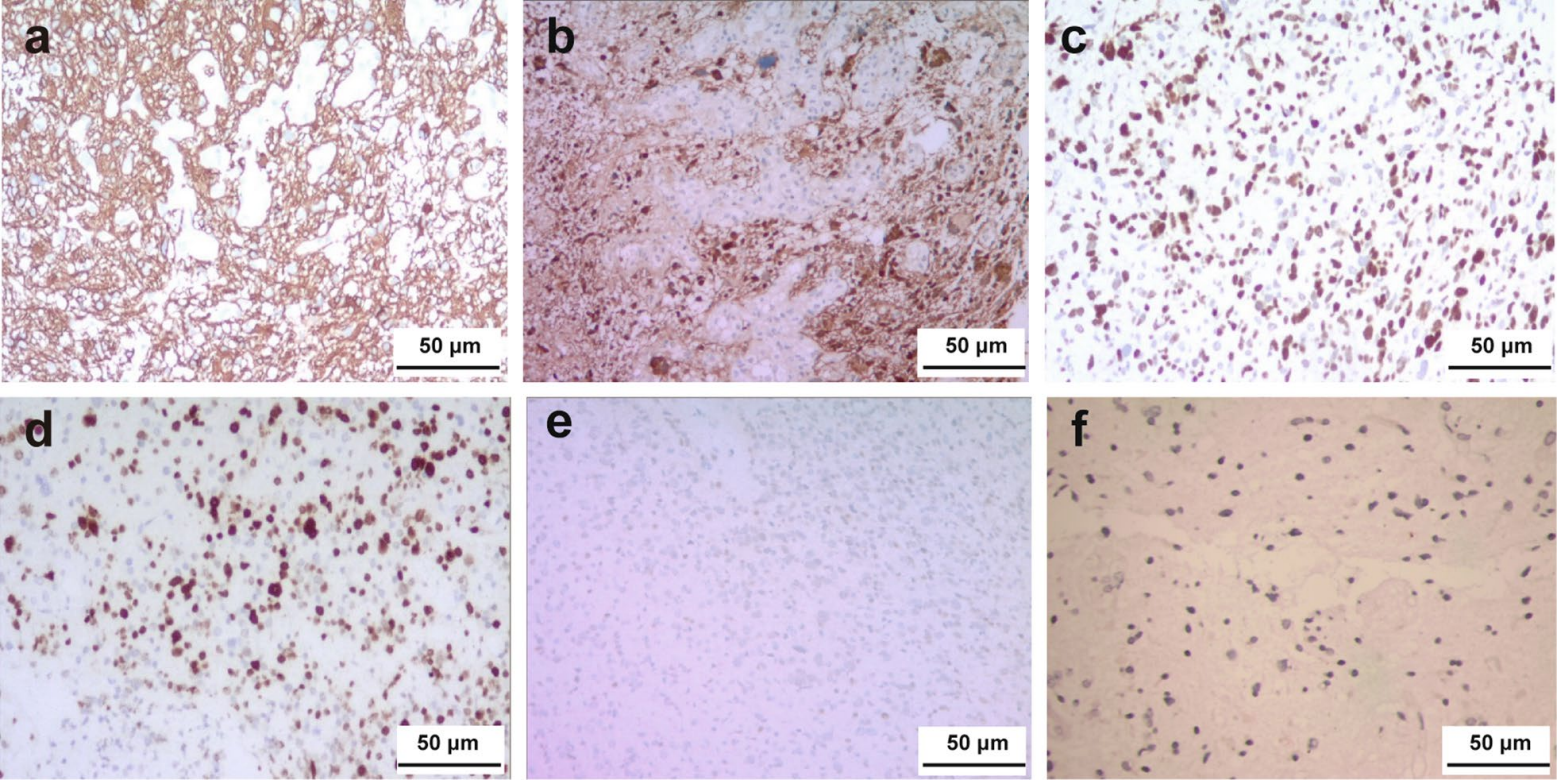
Immunohistochemical examination of the tumor with diaminobenzidine staining. Glial fibrillary acidic protein (**a**), S-100 (**b**), P53 (**c**), and Ki-67 (**d**) are diffusely expressed. The expression of methylated O6-methylguanine-DNA methyltransferase (**e**) and isocitrate dehydrogenase 1 (**f**) is weak

The final pathologic diagnosis was glioblastoma with diffuse and strong expression of glial fibrillary acid protein (GFAP, Fig. 4a) and S-100 (Fig. 4b). The positive rates were 80%–90% for both P53 (Fig. 4c) and Ki-67 (Fig. 4d) and 1% for methylated O6-methylguanine-DNA methyltransferase (Fig. 4e). The expression of isocitrate dehydrogenase 1 (IDH1) was weak (Fig. 4f).

The patient received no further chemotherapy or radiotherapy and died 2 months later.

## Discussion

The preoperative diagnosis of this case was difficult. Brain metastases, central nervous system (CNS) lymphomas, and inflammatory disease were considered in the differential diagnosis. It is notable that the patient presented with frequent fever and cough and had weak positivity for blood TB antibodies. Infectious diseases, such as tuberculosis, were considered. Although previous articles mentioned that ring-like enhancement and multiple lesions are indications of posterior fossa glioblastoma [7], these neurological imaging features are frequently observed in tuberculoma and tuberculous lesions that are occasionally secondary to tuberculous arachnoiditis [8]. Surgical resection or biopsy is necessary for a definitive diagnosis. The histological grading is based on the degree of anaplasia and dedifferentiation, number of mitoses, capillary endothelial proliferation, and presence of necrosis [9–11].

Immunohistochemical examination showed diffuse expression of GFAP, P53, and S-100 in our case. The expression of S-100 is not stable in glioma, whereas GFAP is a qualitative diagnostic marker of glioma [12]. The combination of all histopathologic evidence supported the diagnosis of glioblastoma in our case.

The progressive growth pattern of the CPA lesion is a confusing characteristic of this case, especially given the coexistence of multiple lesions. We searched PubMed and Google Scholar and found only two English-language articles reporting the coexistence of multiple lesions and a CPA lesion [13, 14]. According to the literature, CPA glioblastomas may have different origins [15]. Wu et al. [16] reported a CPA glioblastoma that originated from the cranial VIII nerve and reviewed seven other similar cases. Cranial nerve glioblastomas are presumably originated from CNS tissue locating at the root entry zone of the cranial nerve or from heterotopic neuroglia cells in the leptomeninges. Similarly, Mabray et al. [17] recently reported eight cases of glioblastoma originated from the cranial nerve. These glioblastomas were located at the CPA and involved the unilateral brainstem. The cerebellum is another unusual place for glioblastoma development. Matsuda et al. [18] reported a case of glioblastoma originated from the cerebellum and reviewed another three similar cases. In the reports by both Mabray et al. [17] and Matsuda et al. [18], the relatively clear boundary between the brainstem and tumor were demonstrated during the operation. In our case, neither preoperative MRI nor surgery alone supported an origin at the cranial nerve cerebellum. However, no clear boundary between the CPA lesion and the pons was discovered during our surgical procedure. This evidence, along with MRI findings on the CPA lesions and two pontine lesions, confirmed the diagnosis of primary exophytic pontine glioblastoma. The T2-weighted image showed that the CPA lesion was confined to the pons, with an outer layer that consisted of edematous tumor tissue infiltrating the pons. This finding may explain that the pathologic presentation of the outer layer of the tumor was not as typical for glioblastoma as that of the inner part of the tumor, emphasizing the diverse pathologic features of different parts of the tumor and the need for accurate and comprehensive specimen collection in such a case. As reported earlier, the biopsy samples should be collected from solid, non-necrotic, and gadolinium-enhanced areas, if possible [19].

According to the imaging and histopathologic evidence, this case of pontine glioblastoma can be categorized as a brainstem glioma. However, the classification of brainstem gliomas is not uniform. Choux et al. [20] classified brainstem tumors as diffuse glioma, focal intrinsic glioma (solid or cystic), exophytic glioma, and cervicomedullary glioma. Fisher et al. [21] anatomically classified brainstem glioma as diffuse intrinsic pontine glioma (DIPG), exophytic medullary glioma, and midbrain or tectal glioma. However, they also considered it was reasonable to categorize brainstem tumors simply as diffuse or focal glioma. Our case possessed some typical features of DIPG, such as an infiltrative lesion occupying 50% of the pons, obvious expansion of the pons, and engulfment of the basilar artery as reported in the literature [22, 23]. DIPG may extend laterally into the cerebellar peduncles, cerebellar hemispheres, midbrain, and medulla [24], whereas the exophytic growth to the CPA is not a common feature of typical DIPG. This situation may explain why some researchers exclude exophytic pontine glioma from the diagnostic criteria of DIPG [22, 25]. Given that various manifestations of DIPG are often observed, the term “atypical DIPG” was adopted by some researchers [26]. It seems that our case may be classified as diffuse brainstem (pontine) glioma or an atypical subtype of DIPG (Table 1).

**Table 1:** The features of different types of glioma similar to our case

Tumor type	Symptoms	Location	Distribution	MRI manifestations	Exophytic growth pattern	Relation to BA	MRS	Pathologic subtypes
DIPG	Triad of cerebellar signs, long tract signs, and CN palsy	Intrinsic, central location in the pons	More than 50%–66% of the pons in axial diameter	Heterogeneous, diffuse, and ring-like enhancement	No	Engulfment of BA	With or without elevated Cho:NAA ratio	Astrocytoma, anaplastic astrocytoma, and glioblastoma (majority)
Exophytic pontine glioma	Part of the above triad and headache	The pons	Diffuse	Peripheral or ring-like enhancement	Doral, ventral, left, or right side of the cerebellum, CPA (rarely)	Not mentioned	With or without elevated Cho:NAA ratio	Glioblastoma and low-grade glioma
CPA glioma	CN involvement	The CPA, CN V, and pons	Focal	Irregular or peripheral enhancement	Yes/no	No	With or without elevated Cho:NAA ratio	Glioblastoma, fibrillary astrocytoma, pilocytic astrocytoma, and glioblastoma
Our case	CN involvement	Multiple lesions involving the CPA and pons	Diffuse	Heterogeneous and ring-like enhancement	Yes	Yes	Progressively increasing Cho:NAA ratio	Glioblastoma

*MRI* magnetic resonance imaging, *BA* basilar artery, *MRS* magnetic resonance spectroscopy, *DIPG* diffuse intrinsic pontine glioma, *Cho* choline, *NAA N*-acetyl aspartate, *CPA* cerebellopontine angle, *CN* cranial nerve

The changes in the MRS results of our case merit attention. It was reported that the Cho:NAA ratio calculated according to MRS data can be used to monitor metabolism and glioma behavior [22]. In pediatric CNS tumors, changes in levels of metabolic markers as determined with MRS are associated with tumor grade [27]. Furthermore, an increasing Cho:NAA ratio predicts a short overall survival [22, 28]. PET measures the regional cerebral glucose metabolism, offering valuable information that can be used to differentiate between low-grade and high-grade gliomas [29–31]. For our case, the initial MRS results (obtained on October 8, 2015) showed a simultaneous decrease in NAA and Cho and an increased Cho:NAA ratio, leading to the diagnosis of low-grade glioma. The decrease in NAA, Cho, and Cho:NAA ratio of the first MRS may be technique variations such as the selection bias of regions of interests. Taking both MRS and PET results into consideration, a diagnosis of low-grade glioma seems reasonable. Earlier PET findings from another medical center were consistent with our initial MRS results. Repeated MRS performed 2 and 3 months later demonstrated an obvious decrease in NAA and an increase in Cho, indicating a high-grade glioma. In addition, we noticed peaks at approximately 1.3 ppm (Figs. 1c, 2c, f). Two situations were considered: (1) technical problems such as unstable baseline or lipid effection and (2) a lac-lip peak demonstrating necrosis or hypoxia. Although necrosis is usually found in high-grade glioma, PET–CT results and the increasing tendency of the Cho:NAA ratio both support the initial diagnosis of low-grade glioma. The MRS characteristics of this tumor seemed to be significantly altered within merely 2 months according to consecutive neurological imaging.

Glioblastomas are divided into two types of genesis patterns: primary glioblastoma, also termed de novo glioblastoma, which is defined as a fully developed tumor with no clinical, radiological, or histopathologic evidence of relative benign precursor lesion [32–34], and secondary glioblastoma progressing from low-grade astrocytoma, as supported by imaging or histological evidence [32, 33, 35]. According to the literature, the proportions of primary and secondary glioblastomas are approximately 90% and 10%, respectively [36]; the disease course of primary glioblastomas was less than 3 months, whereas the progression from relative low-grade astrocytoma to glioblastoma took approximately 2–5 years [37]. Nevertheless, rapidly progressive cases of secondary glioblastoma have been reported [34]. Furthermore, as for brainstem gliomas, it has occasionally been reported that low-grade gliomas behaved as aggressively as high-grade gliomas [38, 39]. Recent studies have revealed the histological heterogeneity of DIPG. Based on the autopsy results of the patients diagnosed with DIPG, It was reported that the primary and metastatic lesions displayed high and low histological grades, respectively [40, 41]. Accordingly, DIPG was classified as primary lesions, contiguous lesions, and metastatic lesions according to the locations [41]. The different pathologic manifestations of the two samples from our case may be related to the differences of “central” and “marginal” areas of the lesion. This may again emphasize the importance of accurate sampling.

Immunohistochemical examination of our case demonstrated a high positive rate of P53 (80%–90%), which is a frequently expressed marker of DIPG [42]. It was also reported that P53 mutation was even more frequently and increasingly expressed in secondary glioblastomas, which was in contrast with its rare expression in primary glioblastoma [34]. However, IDH1, a biomarker of secondary glioblastoma [43], showed low expression in our case. This may highlight the molecular differences between pediatric and adult secondary glioblastomas [44–46]. In children, low expression of IDH1 may be present in secondary glioblastomas. According to the literature, histological or imaging evidence of a progression from a less malignant astrocytoma can be used as a diagnostic criterion of secondary glioblastomas [34]. Based on this knowledge and results of repeated MRI (MRS), in combination with initial PET–CT findings, we were inclined to diagnose our case as a rapidly progressive secondary glioblastoma.

Multiple gadolinium-enhanced lesions are rarely seen in brainstem gliomas [47–49]. Multiple-lesion gliomas involve at least two enhanced lesions and account for 2%–9% of all gliomas [50–52]. This rare entity is classified into two distinct types, multifocal and multicentric gliomas. Multifocal gliomas possess homology or dissemination of definite pathways. Multicentric gliomas are defined as anatomically isolated lesions and are usually distributed at different lobes or hemispheres of the brain [51]. Multiple lesions can be present at the diagnosis or at a progressive stage for both multicentric and multifocal gliomas [53]. In our case, the right pontine lesion was developed at a later stage as compared with the other two lesions, and all lesions were located at the diffusely swelling pons, indicating a diagnosis of multifocal glioblastoma rather than multicentric glioblastoma.

Diffuse pontine glioma is the most common type of brainstem tumor and carries the poorest prognosis in children [23]. Early surgical intervention to determine the diagnosis is recommended [54–56]. The standard treatment of DIPG is external beam radiation therapy administered in fractions over approximately 6 weeks at a total dose of 60 Gy [54]. Diffuse intrinsic pontine glioma is almost invariably fatal with a mean patient overall survival of 9–12 months from the time of diagnosis [57]. Radiation therapy is the major treatment [58]. Adjuvant therapies, such as chemotherapy, targeted therapy, differentiation agents, and radiation sensitizers, have been studied. Although some of these therapies showed a beneficial impact on patient outcomes [25, 58], further research is required.

## Conclusion

In summary, we presented a rare case of progressive, exophytic, multifocal pontine glioblastoma in a 5-year-old girl. A preoperative differential diagnosis was difficult because of the atypical neurological imaging features and frequent fever and cough symptoms. The clinical evidence supported a secondary glioblastoma. Regretfully, an early histopathologic diagnosis was not achieved.
